# Collapsing factors in multitrait-multimethod models: examining consequences of a mismatch between measurement design and model

**DOI:** 10.3389/fpsyg.2015.00946

**Published:** 2015-08-03

**Authors:** Christian Geiser, Jacob Bishop, Ginger Lockhart

**Affiliations:** Department of Psychology, Utah State UniversityLogan, UT, USA

**Keywords:** multitrait-multimethod (MTMM) analysis, confirmatory factor models, method factors, estimation problems, negative variances, interchangeable versus structurally different methods, bifactor models, latent state-trait models

## Abstract

Models of confirmatory factor analysis (CFA) are frequently applied to examine the convergent validity of scores obtained from multiple raters or methods in so-called multitrait-multimethod (MTMM) investigations. Many applications of CFA-MTMM and similarly structured models result in solutions in which at least one method (or specific) factor shows non-significant loading or variance estimates. Eid et al. (2008) distinguished between MTMM measurement designs with interchangeable (randomly selected) vs. structurally different (fixed) methods and showed that each type of measurement design implies specific CFA-MTMM measurement models. In the current study, we hypothesized that some of the problems that are commonly seen in applications of CFA-MTMM models may be due to a mismatch between the underlying measurement design and fitted models. Using simulations, we found that models with *M* method factors (where *M* is the total number of methods) and unconstrained loadings led to a higher proportion of solutions in which at least one method factor became empirically unstable when these models were fit to data generated from structurally different methods. The simulations also revealed that commonly used model goodness-of-fit criteria frequently failed to identify incorrectly specified CFA-MTMM models. We discuss implications of these findings for other complex CFA models in which similar issues occur, including nested (bifactor) and latent state-trait models.

## Introduction

Multitrait-multimethod (MTMM) analysis (Campbell and Fiske, 1959) is a popular approach for examining the convergent and discriminant validity of psychological measurements based on measurement designs in which multiple constructs or traits are assessed by multiple methods (Widaman, 1985; Millsap, 1995; Dumenci, 2000). The analysis of MTMM data has historically focused on the interpretation of the so-called MTMM matrix, which contains the correlations between observed variables in an MTMM design. The MTMM matrix approach was developed by Campbell and Fiske (1959) who also proposed heuristics for the interpretation of MTMM correlations in terms of convergent and discriminant validity. Over the years, confirmatory factor analysis (CFA) has become a popular tool for analyzing data obtained from MTMM designs, given the greater flexibility of the CFA framework compared to the original MTMM matrix approach (for detailed discussions of the advantages of the CFA approach to MTMM analyses, see Eid et al., 2003, 2006 as well as Marsh and Hocevar, 1988).

Despite the fact that CFA has proven to be a valuable tool for analyzing MTMM data and is widely used for this purpose, empirical applications of CFA-MTMM models are not always free of problems. In fact, CFA-MTMM applications are often plagued by convergence problems and improper solutions (e.g., zero or negative latent variance estimates; e.g., Marsh, 1989; Marsh and Bailey, 1991; Kenny and Kashy, 1992; Lance et al., 2002). In the present study, we focused on a specific problem that is frequently encountered in CFA-MTMM studies: The problem of one or more method factors collapsing (i.e., showing non-significant loadings and/or variance estimates). We hypothesized that this problem may be related to a mismatch between the measurement design (i.e., the types of methods used in the study) and the CFA measurement model chosen to analyze the data. Building on Eid et al.'s (2008) theoretical distinction between *interchangeable* and *structurally different methods*, we used simulations to examine consequences of applying models that are designed for interchangeable methods to data generated by structurally different methods and vice versa.

Our paper is organized as follows. We first provide a review of Eid et al.'s (2008) theoretical framework for distinguishing measurement designs with interchangeable vs. structurally different methods and corresponding CFA-MTMM models. We then show that a number of empirical studies that used CFA-MTMM or similarly structured models found at least one method factor to be unstable and explain why this problem may be related to a mismatch between measurement design and CFA-MTMM models. Subsequently, we present the results of a simulation study in which we investigated the potential consequences of such a mismatch for the estimation of method factors. In our Discussion section, we highlight implications for MTMM and other complex CFA models, such as latent state-trait (LST) models (Steyer et al., 1992) and nested (bi)factor models (e.g., Reise, 2012).

### Theoretical framework: Interchangeable vs. structurally different methods

Eid et al. (2008) distinguished between MTMM measurement designs with interchangeable methods and MTMM measurement designs with structurally different methods (as well as the combination of both types of methods)^1^. Eid et al. (2008) showed that from a measurement theoretical perspective, this distinction has implications for which CFA-MTMM measurement model is most suitable in a given application. Interchangeable (or random) methods are methods that are randomly selected from a universe of equivalent methods (e.g., randomly selected students rating university professors' teaching abilities).

In contrast, structurally different methods are fixed and non-interchangeable. As an example, consider self, parent, and sibling reports chosen as methods to rate individuals' depression levels. For each target individual, his or her parents and siblings (as well as the self-report) are fixed and cannot be replaced by another draw from a universe of parent or sibling reports. Moreover, self, parent, and sibling reports each provide potentially unique, non-overlapping information about the targets such that, for example, a parent report could not be replaced by the corresponding sibling report without a potential loss of information.

Eid et al. (2008) as well as Koch et al. (2014) showed that from a measurement theoretical perspective, each type of method (interchangeable vs. structurally different) implies a distinct type of random sampling experiment. In the case of interchangeable methods, we deal with a two-stage sampling procedure. In the first step, we select an (ideally) random sample of targets (e.g., a random sample of teachers for whom we want to measure teaching quality). In the second step, we (ideally) randomly select methods (e.g., test items or student raters) from the universe of available methods for the given targets. In contrast, with structurally different methods, only the targets are (ideally) sampled at random, whereas the methods are fixed given the targets. For example, once we identify a given sample of professors, their self- and department head reports of teaching quality are fixed (whereas we could draw a random sample of students or peers to rate the professors, which could then we seen as interchangeable methods). The two-stage sampling design that is employed with interchangeable methods implies a nested or multilevel structure, whereas an MTMM design with only structurally different (fixed) methods does not.

According to Eid et al. (2008) the different types of sampling procedures have logical implications for which type of CFA-MTMM model should be used to analyze the data. In other words, interchangeable methods call for a different type of model than structurally different methods. This parallels the use of different models for random vs. fixed factors in analysis of variance.

#### CFA-MTMM model for interchangeable methods

Eid et al. (2008) showed that given the two-stage sampling procedure, measurement designs with interchangeable methods imply a data structure with methods nested within targets. Nussbeck et al. (2009) presented a single-level CFA-MTMM model for interchangeable methods, which we used in the present study. This model is similar in structure compared to LST (Steyer et al., 1992) and bifactor (e.g., Reise, 2012) models, but with specific parameter equality constraints as described below.

**Figure 1:**
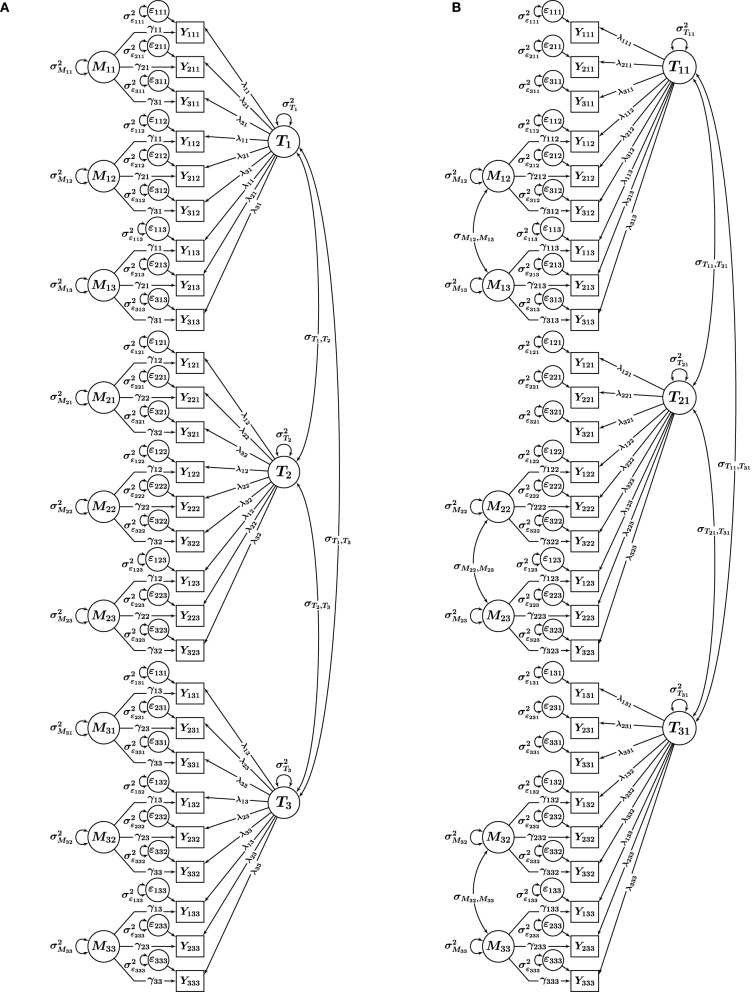
**CFA-MTMM models for a design with *i* = 1, 2, 3 indicators, *t* = 1, 2, 3 traits, and *m* = 1, 2, 3 methods**. **(A)** CT-UM_constrained_, **(B)** CT-C (*M*−1). *Y*_*itm*_, observed variables; *T*_*t*_, *T*_*tm*_, trait factors; *M*_*tm*_, trait-specific method factors; ϵ_*itm*_, measurement error variables; λ_*it*_, λ_*itm*_, trait factor loadings; γ_*it*_, γ_*itm*_, method factor loadings.

Nussbeck et al.'s (2009) CFA-MTMM model for interchangeable methods is illustrated in Figure 1A for three traits (*t* = 1, 2, 3) measured by three methods (*m* = 1, 2, 3) using three indicators (observed variables *Y*_*itm*_, where *i* = 1, 2, 3 indicates a specific item or scale) per trait-method combination (TMC) (Note that throughout this paper, we assume that *multiple* indicators *i* are available for each TMC, given the advantages of multiple-indicator models noted by Marsh and Hocevar, 1988). It can be seen that the model contains a trait factor *T*_*t*_ for each trait and a (trait-specific) method factor *M*_*tm*_ for each interchangeable method. Trait and method factors are uncorrelated by definition. Furthermore, method factors have means of zero by definition and are assumed to be uncorrelated with each other, given the assumption of randomly selected methods. Given these properties and in order to be consistent with the general literature on CFA-MTMM models, we refer to this model as the correlated traits-uncorrelated methods constrained (CT − UM_constrained_) model.

A special feature of the CT − UM_constrained_ model is the implicit assumption of invariant measurement parameters across interchangeable methods. For this reason, we denote this model using the subscript constrained. This assumption parallels the assumption of measurement equivalence (or invariance) often made in multigroup and longitudinal analyses and means that the intercepts as well as the trait and method factor loadings are assumed to be constant across methods for the same indicator within a given trait *t*. These formal parameter invariance constraints reflect the expectation that truly interchangeable methods should not differ in their relation to the trait factor (Geiser et al., 2014a). The assumption of invariant loadings and intercepts is illustrated in Figure 1A in terms of the intercept and loading parameters carrying indices only for the measured variable (*i*) and the trait (*t*), but not for the method (*m*).

In more restricted versions of the model, in addition to the intercepts and factor loadings, also the measurement error and method factor variances may be assumed to be invariant across interchangeable methods. Measurement invariance (MI) assumptions may be violated (and falsified through model goodness-of-fit tests) in empirical applications if methods were not truly selected at random from a pool of interchangeable methods. Testing for MI thus represents an empirical check of whether the methods used in a study can be viewed as interchangeable or whether they should rather be treated as structurally different (Geiser et al., 2014a)^2^.

#### CFA-MTMM model for structurally different methods

For structurally different methods, Eid et al. (2008) recommended the use of a modeling approach that contrasts *M*−1 so-called non-reference methods against a reference method (where *M* denotes the total number of structurally different methods used in the study). This approach was originally presented by Eid (2000) and specifies *M*−1 correlated residual method factors, that is, a method factor for each non-reference method. No method factor is specified for the reference method. The reference method is typically selected based on a researcher's theory about which method may be most valid for assessing the construct (e.g., a psychometric intelligence test would likely be seen as a gold standard measure and as more valid for measuring intelligence than a self-rating of a person's IQ score), which method may have the most immediate access to the constructs of interest (e.g., self-reports of certain types of emotions may be more relevant than other reports, because emotions may be covert and not easily accessible by external observers), or which method is most outstanding or special (e.g., cortisol measurements of stress vs. self- and other ratings). In cases in which there is little or no theory about the most valid method, researchers typically choose a method to serve as reference that is most unique relative to the remaining methods (e.g., behavioral observations by experts vs. self-, parent, or teacher-reports of behavior).

In Figure 1B, Eid et al.'s (2008) correlated traits-correlated (methods minus one) or CT − C(*M* − 1) model is illustrated for a design with three traits and three structurally different methods. In this example, without loss of generality, Method 1 (*m* = 1) serves as reference method. This can be seen from the fact that there is no method factor for this method. Therefore, the trait factors *T*_*t*1_ are specific to the reference method. We make this clear by using two indices for the traits in Figure 1B (one for the trait and one for the reference method [*m* = 1]). The indicators of the remaining (non-reference) methods load onto the reference trait factor and onto (trait-specific) residual method factors *M*_*tm*_. Being defined as residuals with respect to the traits, the method factors have means of zero and are uncorrelated with the trait factor pertaining to the same trait [i.e., cov(*T*_*t*1_, *M*_*tm*_) = 0].

Eid et al. (2008) recommended the use of the CT − C(*M* − 1) approach for structurally different methods, because this approach is in line with the sampling procedure implied by structurally different methods. With structurally different methods, each type of structurally different method represents a unique perspective or “fixed effect.” The underlying sampling procedure implies that structurally different methods are “at the same level” with the targets and not nested within the targets (as is the case with interchangeable methods). A meaningful way to compare structurally different methods is thus to contrast them against a reference method, as is done in the CT − C(*M* − 1) approach (Geiser et al., 2008, 2014b). This parallels what is commonly done in regression analysis with fixed categorical predictors, where researchers may use *K* − 1 dummy code variables to represent the *K* levels of a fixed factor.

### Empirical practice and problems in CFA-MTMM analyses

**Figure 2:**
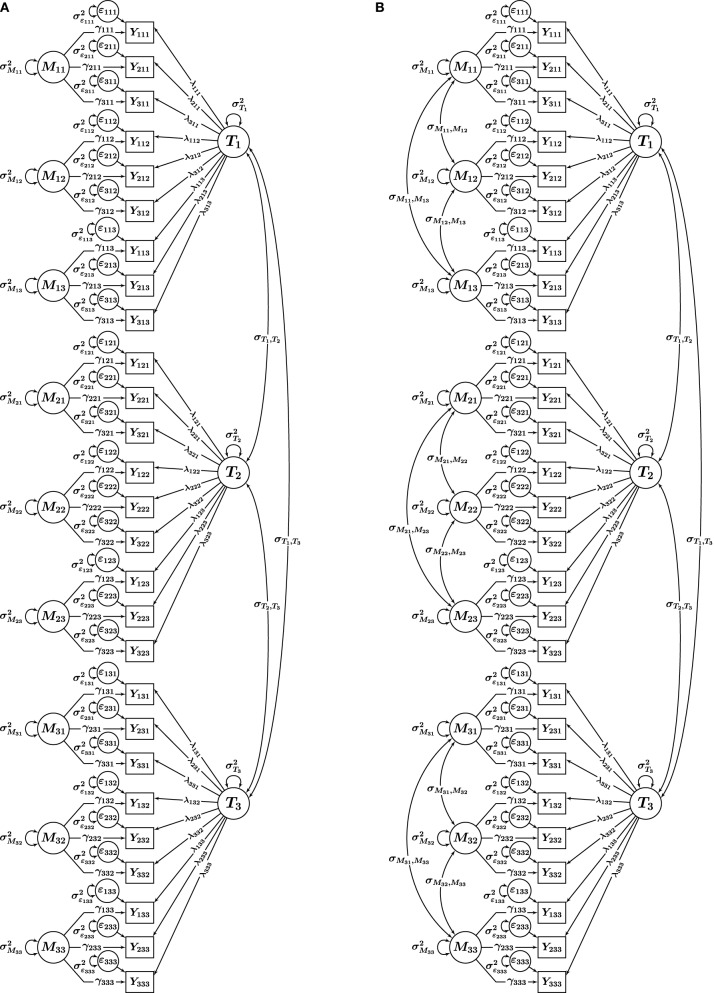
**CFA-MTMM models for a design with *i* = 1, 2, 3 indicators, *t* = 1, 2, 3 traits, and *m* = 1, 2, 3 methods**. **(A)** CT-UM_unconstrained_. **(B)** CT-CM. *Y*_*itm*_, observed variables; *T*_*t*_, trait factors; *M*_*tm*_, trait-specific method factors; ϵ_*itm*_, measurement error variables; λ_*itm*_, trait factor loadings; γ_*itm*_, method factor loadings.

Our key consideration in the present article is that in practical applications, researchers frequently use different CFA-MTMM models without explicitly considering the type of measurement design (interchangeable vs. structurally different methods). Researchers in the past have applied models that resemble Nussbeck et al.'s (2009) model for interchangeable methods to data that were either clearly or very likely generated by structurally different (rather than interchangeable) methods. In particular, many applications have used a CT-UM model with unconstrained factor loadings, which we refer to as correlated traits-uncorrelated methods [CT − UM_unconstrained_] approach (e.g., Marsh and Grayson, 1995). The CT − UM_unconstrained_ model (see Figure 2A) closely resembles the model in Figure 1A (correlated trait factors and *M* uncorrelated method factors), but with factor loadings not set equal for the same indicators across methods. We use the subscript unconstrained to distinguish this model from Nussbeck et al.'s (2009) CT − UM_constrained_ model.

Furthermore, it is common to use CFA-MTMM models with *M* correlated method factors for structurally different methods, and this practice has recently been advocated by methodological researchers (Lance et al., 2002). This approach is known as the correlated traits-correlated methods (CT − CM) approach and is illustrated in Figure 2B. In the present study, we tested the hypothesis that some of the commonly seen problems with the CT − UM_unconstrained_ and CT − CM models occur more frequently when these models are applied to data generated from structurally different methods. We suspected that this problem may in part be obscured by the fact that factor loadings are typically not constrained in empirical applications of the CT − UM_unconstrained_ and CT − CM models, leading to seemingly well-fitting models. That is, we hypothesized that the CT − UM_unconstrained_ and CT − CM models with unconstrained loadings as highly parameterized models would fit a wide variety of data (i.e., from both interchangeable and structurally different methods) well, yet produce a higher rate of abnormal results.

It is well-known that many applications of the CT − UM_unconstrained_ and especially CT − CM model in the past have led to estimation problems such as non-convergence or improper parameter estimates. This problem has occurred both in empirical and simulated data with correctly specified models (Marsh, 1989; Castro-Schilo et al., 2013). A particularly interesting phenomenon is the fact that a substantial portion of empirical applications of models similar in structure to the CT − UM_unconstrained_ and CT − CM models with either MTMM and other similarly structured data have resulted in one or more method factors showing non-significant variance estimates or non-significant loadings for the majority or even all indicators of these factor(s). We refer to this issue as the “method factor collapse” problem, as it implies that the existence of the method factor(s) in question is not empirically supported.

For example, Maydeu-Olivares and Coffman (2006) applied the CT − UM_unconstrained_ model (termed bifactor model by these authors) to positively worded items (Method 1) and negatively worded items (Method 2) of a scale for measuring the trait *optimism*. Maydeu-Olivares and Coffman (2006) found that three out of four loadings on the method factor for the negatively worded items were not significantly different from zero. Chen et al. (2006) fit a bifactor model to different domains of quality of life and found that four out of five loadings on their specific (“method”) factor representing the mental health facet of quality of life were not statistically significant. In addition, Chen et al. (2006) reported that the specific factor in question had a negative and non-significant latent variance estimate and that three out of five factor loadings on this factor were negative, which was contrary to the a priori hypotheses made by these authors. This finding led Chen et al. (2006) to drop the specific factor for mental health from their model, resulting in a model respecification with *M*−1 (rather than the originally assumed *M*) specific factors. Similarly, Chen et al. (2012) found non-significant variances of the specific factor “Warmth” in two applications of the bifactor model to the measurement of extraversion, subsequently dropping this factor from the model. Another example of a weak specific factor in an empirical application can be found in Holzinger and Swineford's (1937) classical article on the bifactor model. Schermelleh-Engel et al. (2004) noted that the finding of one of *M* method (or specific) factors collapsing is also common in applications of LST models (Steyer et al., 1992), which are similar in structure to CT-UM and bifactor models.

The issue of a method or specific factor collapsing can cause problems in empirical applications, because the meaning and interpretation of the trait factor changes in this event. The trait factor no longer represents a general trait, but becomes specific to the method(s) or facets for which the method or specific factor(s) collapsed. For example, in Chen et al.'s (2006) quality of life application, the general trait factor after dropping the specific factor for “mental health” becomes the common true score of the mental health indicators. It is thus no longer a *general* quality of life factor, but instead becomes a specific “mental health” factor. Hence, the interpretation of the trait factor would be similar to the interpretation in the CT − C(*M* − 1) approach, in which this factor represents the common true score variable of the indicators pertaining to the reference method. The difference, however, is that in the method factor collapse case, the “reference method” is not chosen *a priori* by the researcher based on theory, but is data-driven. The *post-hoc* interpretation of the trait factor is not the same as the *a priori* hypothesized or intended interpretation. Among other issues, this may lead to the question of whether this empirical result is arbitrary or whether it generalizes across studies. Although not all applications of CT − UM_unconstrained_, LST, or bifactor models show the problem of one or more specific (method) factors collapsing empirically, in our view, this issue is nonetheless remarkable and worthy of further study, given that this problem seems to occur in a significant portion of applications of models with a CT − UM_unconstrained_-type structure and leads to complications in the interpretation of the latent factors in the models.

In summary, in the present study, we hypothesized that the problem of one method factor collapsing (or becoming empirically unstable) in applications of models with *M* (rather than *M*−1) method factors may be related to the fact that researchers typically analyze structurally different (fixed) methods (e.g., different raters or structurally different, non-interchangeable facets of a construct such as quality of life or intelligence). Based on Eid et al.'s (2008) measurement theory of interchangeable vs. structurally different methods, we expected that the use of *M* (correlated or uncorrelated) method factors with unconstrained factor loadings for structurally different methods would result in generally well-fitting models, but with a higher rate of empirically unstable method factors. This hypothesis was based on the fact that according to Eid et al.'s (2008) framework, models with *M* (rather than *M*−1) method factors and free loadings overfit data generated by structurally different methods. We tested our hypotheses in a simulation study of a simplified (single-trait) MTMM design as described below.

## Methods

In our simulation, we examined designs with just a single trait, but three methods and three indicators per TMC. We focused on this simplified design because (1) it is sufficient to examine the hypothesized method factor instability issue and (2) models with a single trait factor and three or more specific method factors are frequently used in empirical studies [e.g., bifactor models in intelligence research (Schmiedek and Li, 2004) and LST models for longitudinal data analysis (Steyer et al., 1992)]. Since these models use only a single trait, there were no correlated traits. Therefore, for simplicity, we subsequently dropped the trait index t as well as *CT* from each model name.

**Figure 3:**
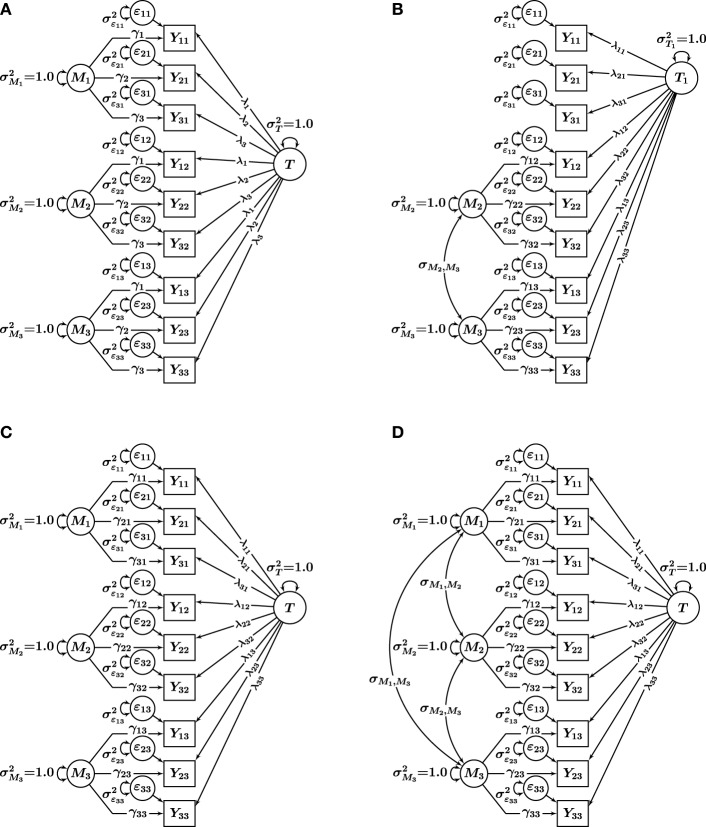
**Single-trait multi-method models used in the simulation**. **(A)** UM_constrained_ model for interchangeable methods. **(B)** C (*M*−1) model for structurally different methods. **(C)** UM_unconstrained_ model, and **(D)** CM model. *i* = 1, 2, 3 indicators, and *m* = 1, 2, 3 methods. *T*, *T*_*m*_, trait factors; *M*_*m*_, method factors; ϵ_*im*_, measurement error variables; λ_*im*_, trait factor loadings; γ_*im*_, method factor loadings.

Based on Eid et al.'s (2008) MTMM measurement theory, we generated data using (1) the UM_constrained_ model (interchangeable methods case) and (2) the C(*M* − 1) model (structurally different methods case). The two population models are shown in Figures 3A,B. Four models were fit to each set of population data: (1) UM_constrained_, (2) C(*M* − 1), (3) UM_unconstrained_, and (4) CM. The four fitted models are shown in Figures 3A–D. The simulation thus included both conditions of *correctly specified models* [UM_constrained_ data fit with a UM_constrained_ model; C(*M* − 1) data fit with a C(*M* − 1) model] and *incorrectly specified models* [UM_constrained_ data fit with UM_unconstrained_, C(*M* − 1), and CM models; C(*M* − 1) data fit with UM_constrained_, UM_unconstrained_, and CM models].

We included sample size and population parameter conditions that are likely to be found in empirical applications. We simulated two sample sizes (250, 1000), nine levels of consistency (true score variance in an indicator that is explained by the trait factor; 0.1, 0.2, 0.3, 0.4, 0.5, 0.6, 0.7, 0.8, 0.9), and three levels for indicator reliability (0.6, 0.7, 0.8). In addition, the latent correlation between the two method factors in the population C(*M* − 1) model was varied using three different levels (0.2, 0.5, 0.8; for the UM_constrained_ model all method factor correlations are 0 by definition). The size of this simulation was

$$\begin{array}{l} \text{thus }[\underset{\text{Pop}.\text{ Model}:\;{\text{UM}}_{\text{constrained}}}{\underset{︸}{\left(2\times 9\times 3\right)}}\times \underset{\text{corr}(M_{2},M_{3})}{\underset{︸}{1}}+\underset{\text{Pop}.\text{ Model}:\text{ C}(M-1)}{\underset{︸}{\left(2\times 9\times 3\right)}}\\ \;\times \underset{\text{corr}(M_{2},M_{3})}{\underset{︸}{3}}]\;\times \underset{\text{Fit Model}}{\underset{︸}{4}}=864\text{ cells}. \end{array}$$

### Population parameters and sampling

There were two main steps involved in developing the simulation inputs: (a) specifying model parameters and sampling from the population, and (b) specifying the models. Population parameters were defined using the parameter levels shown in Table 1. Note that a small amount of variability was allowed in the indicator reliabilities to obtain a more realistic scenario^3^. The implied mathematical relations among model parameters, indicator reliability, consistency, and total observed variance given in Table 1 were then used to determine the true values for remaining population parameters. No mean structure was included in any of the models. After the population parameters were chosen, the Monte Carlo facility of the Mplus software (Muthén and Muthén, 1998–2012) was used to randomly draw 1000 samples from each cell of the design assuming complete multivariate normal data. Each set of sample data was saved to a separate file.

**Table 1:** **Simulation parameters**.

**Parameter (abbreviation)**	**Levels**	**Description**
Population model	{UM_constrained_, C(*M* − 1)}	UM_constrained_: Model for interchangeable raters;*C*(*M* − 1): Model for structurally different raters.
Sample size (N)	{250, 1000}	
Consistency (con)	{.2, 0.3, 0.4, 0.5, 0.6, 0.7, 0.8, 0.9}	${}^{\text{a}}\;{\text{con}}_{im}=\lambda_{im}^{2}\text{ var}(T_{t})\lambda_{im}^{2}\text{ var}(T_{t})+\gamma_{im}^{2}\text{ var}(M_{m})$
Non-reference		
method correlation	{0, 0.2, 0.5, 0.8}	corr(*M*_2_, *M*_3_); 0 for UM_constrained_, non-zero for C(*M* − 1).
Reliability	{0.6, 0.7, 0.8} + ~ (β_3, 3_ − 0.5)0.05	$\overset{n}{\underset{i=1}{\sum }}\overset{l}{\underset{m=1}{\sum }}\left[\mathrm{rel}\left(Y_{im}\right)\right]n\cdot l.$
	≈{0.6, 0.7, 0.8}	$\mathrm{rel}\left(Y_{im}\right)=\lambda_{im}^{2}\mathrm{var}\left(T_{t}\right)+\gamma_{im}^{2}\mathrm{var}\left(M_{m}\right)\mathrm{var}\left(Y_{im}\right)$
Trait variance	{1.0}	var(*T*_*t*_)
Method variance	{1.0}	var(*M*_*m*_) for all *m*
Indicator variance	{1.0}	var(*Y*_*im*_) for all *i, m*

*Index i = 1…n indicates the manifest variable or indicator. Index m = 1…l indicates the method. β_3, 3_ refers to the beta distribution with shape parameters 3,3. ^a^Consistency for the first indicator in the C(M − 1) model is defined as being equal to the reliability for that indicator, which implies no method variance for the reference (“gold standard”) method*.

### Model specification

Again using Mplus, all four models were fit separately to each of the data files using maximum likelihood estimation. For identification, latent trait and latent method factor variances were fixed to 1.0 in all models [i.e., var(*T*) = var(*M*_*m*_) = 1 for all *m*] in accordance with the specification of the population models; all other parameters were freely estimated, except that the appropriate equality constraints were imposed on the loadings in the UM_constrained_ model and that method factor correlations were only estimated in the C(*M* − 1) and CM models. In each analysis, we allowed a maximum of 1000 iterations. Models were classified as “non-converged” if convergence was not reached after 1000 iterations. The total number of models analyzed in this simulation was 864,000.

### Criteria for evaluating the performance of the models

Four simulation outcomes were examined: (1) non-convergence, (2) improper solutions, (3) goodness of fit, (4) non-significant method factor loadings and factor collapse. These outcomes are discussed in detail below. Regression analysis was used to determine simulation conditions (independent variables) over which results could be aggregated, while ensuring that the main results were still accurately represented.

#### Non-convergence

We recorded the number of replications for which the estimation process did not converge after 1000 iterations. The percent convergence was then computed, which is given by

$$\%\text{convergence}=\frac{\begin{array}{c} \text{replications converged after}\\ \text{1000 iterations} \end{array}}{\text{replications requested}}\times 100.$$

#### Improper solutions

We distinguished between two types of improper solutions. The first type refers to the number of replications with a non-positive definite (npd) residual covariance matrix, Θ (theta). The second type refers to the number of replications with a npd latent variable covariance matrix, Ψ (psi). Percentages based on each of these errors are given by

$$\begin{array}{l} \%\Theta =\frac{\text{number of }\Theta \text{ errors after }1000\text{ iterations}}{\text{number of replications requested}}\;\times \;100,\\ \%\Psi =\frac{\text{number of }\Psi \text{ errors after }1000\text{ iterations}}{\text{number of replications requested}}\;\times \;100. \end{array}$$

#### Model fit

Several fit statistics were examined to determine whether incorrectly specified models could be reliably identified based on commonly used model goodness-of-fit criteria. Following Hu and Bentler (1999) as well as Schermelleh-Engel et al. (2003), the criteria used for appropriate fit were: a non-significant χ^2^ test statistic (i.e., a χ^2^ *p*-value ≥0.05), root mean square error of approximation (RMSEA) ≤0.05, comparative fit index (CFI) ≥0.95, and standardized root mean square residual (SRMR) ≤0.05.

#### Non-significant factor loadings and factor collapse

Non-significant method factor loadings were defined as factor loadings for which the 95% confidence interval for a given factor loading contained 0. We defined factor collapse as a condition in which *all* factor loadings (three for this simulation) for a given method factor were jointly non-significant. Our rationale for this was that in the case in which a method factor does not show a single significant loading, that factor's interpretation would be jeopardized. As discussed previously, the condition of one method factor collapsing empirically in this manner is frequently encountered in practical applications.

## Results

### Non-convergence

The total number of non-converged replications was 90,325 which represented 10.45% of the 864,000 total replications requested. The convergence rates for correctly specified models were quite high, over 99% for both the UM_constrained_ and C(*M* − 1) models. Convergence rates for incorrectly specified models were also high (above 90%), except for the CM model, which showed convergence rates of about 65%. Table 2 shows the rates of non-convergence and improper solutions for each fitted model.

**Table 2:** **Results for convergence and improper solutions**.

**Criteria**	**Population model**	**Fitted model**	**Problematic replications**	**Total replications**	**Percent problematic**
**NON-CONVERGENCE**					
Properly specified					
	UM_constrained_	UM_constrained_	3	54,000	0.01
	C(*M* − 1)	C(*M* − 1)	1,259	162,000	0.78
Misspecified					
	UM_constrained_	C(*M* − 1)	51	54,000	0.09
	UM_constrained_	UM_unconstrained_	1,945	54,000	3.60
	UM_constrained_	CM	19,302	54,000	35.74
	C(*M* − 1)	UM_constrained_	433	162,000	0.27
	C(*M* − 1)	UM_unconstrained_	12,217	162,000	7.54
	C(*M* − 1)	CM	55,115	162,000	34.02
	Total		90,325	864,000	10.45
**IMPROPER SOLUTIONS**					
**Θ errors**					
Properly specified					
	UM_constrained_	UM_constrained_	5	54,000	0.01
	C(*M* − 1)	C(*M* − 1)	709	162,000	0.43
Misspecified					
	UM_constrained_	C(*M* − 1)	48	54,000	0.09
	UM_constrained_	UM_unconstrained_	1,606	54,000	2.97
	UM_constrained_	CM	12,182	54,000	22.56
	C(*M* − 1)	UM_constrained_	293	162,000	0.18
	C(*M* − 1)	UM_unconstrained_	12,053	162,000	7.44
	C(*M* − 1)	CM	49,643	162,000	30.6
	Total		76,539	864,000	8.86
**Ψ errors**					
Properly specified					
	UM_constrained_	UM_constrained_	0	54,000	0.00
	C(*M* − 1)	C(*M* − 1)	554	162,000	0.34
Misspecified					
	UM_constrained_	C(*M* − 1)	2	54,000	0.00
	UM_constrained_	UM_unconstrained_	0	54,000	0.00
	UM_constrained_	CM	5,717	54,000	10.59
	C(*M* − 1)	UM_constrained_	0	162,000	0.00
	C(*M* − 1)	UM_unconstrained_	0	162,000	0.00
	C(*M* − 1)	CM	14,218	162,000	8.78
	Total		20,491	864,000	2.32

*Θ errors = number of replications with an improper residual covariance matrix. Ψ errors = number of replications with an improper latent variable covariance matrix. Problematic replications = number of replications for which non-convergence, Θ errors, or Ψ errors occurred. Total replications = total number of replications for the given model condition*.

### Improper solutions

The total number of improper solutions due to npd Θ matrices was 76,539, which is approximately 9%. The overall number of solutions with npd Ψ matrices was much lower (20,491 or 2%). The rate of both Θ and Ψ warnings for correctly specified models was less than 0.5%. Among the misspecified models, the CM model showed the most Θ and Ψ warning messages (rates between 7.44 and 22.56%). Improper solutions for the other misspecification conditions were less common (below 3%).

### Model fit

**Figure 4:**
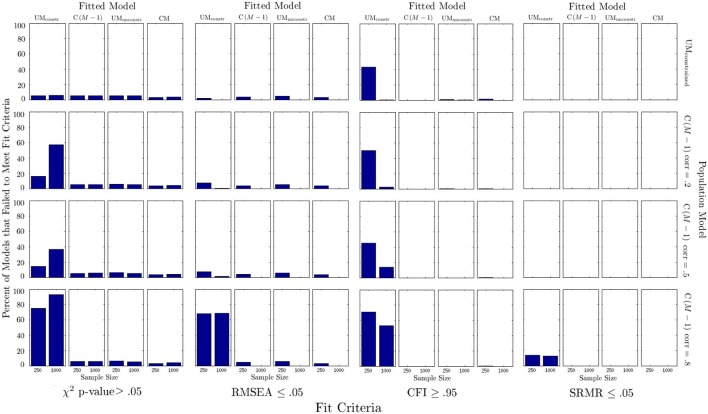
**Percentage of models that were rejected according to different fit criteria**.

Figure 4 shows the percentage of models that would have been rejected according to commonly used model fit criteria. The following observations can be made from Figure 4: (1) Model fit based on χ^2^, RMSEA, and SRMR was generally indicated to be good for all correctly specified models (as it should be); (2) based on the common criterion of 0.95, the CFI would have often mistakenly “rejected” the correctly specified UM_constrained_ model (43% of the time for a sample size of 250); (3) when the UM_unconstrained_ or CM models were fitted to any set of population data these models showed acceptable model fit according to all indices, despite being misspecified; (4) when using conventional criteria, fit indices typically did not indicate when a C(*M* − 1) model was incorrectly fit to data generated from a UM_constrained_ model; (5) when the UM_constrained_ model was fitted to data generated from a C(*M* − 1) model, the χ^2^ test and CFI were often indicating this misspecification (this was the only condition in which incorrectly specified models could have relatively frequently been correctly rejected based on model fit criteria); in this condition, the χ^2^ test was the most reliable indicator of model misfit; the CFI criterion revealed this misspecification relatively frequently in the smaller sample size condition (*N* = 250); the RMSEA criterion resulted in a high rate of correct rejections only for the high method factor correlation condition (*r* = 0.8). Overall, fit indices correctly identified that the UM_constrained_ model was incorrectly fit to C(*M* − 1) data most often when the population method factor correlation was high (0.8) in the data-generating C(*M* − 1) model.

### Non-significant factor loadings and factor collapse

**Figure 5:**
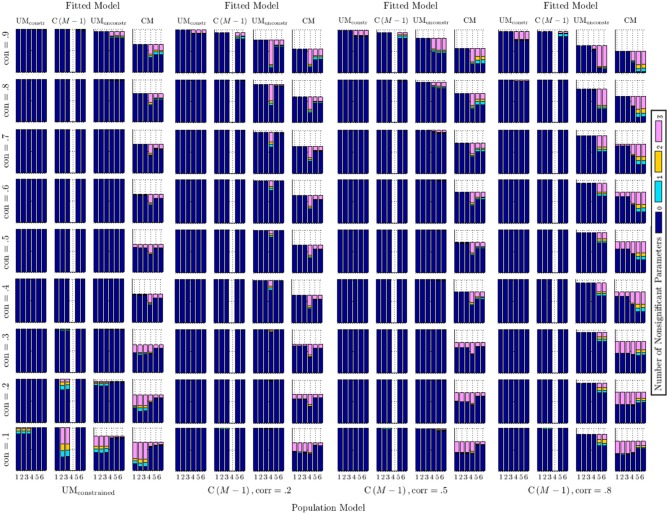
**Number of non-significant method factor loadings across simulation conditions**. Parameters are shown in sets, where Sets 1–3 refer to trait factor loadings and Sets 4–6 refer to method factor loadings. Set 1: λ_11−31_, Set 2: λ_12−32_, Set 3: λ_13−33_, Set 4: γ_11−31_, Set 5: γ_12−32_, Set 6: γ_13−33_. Note that the loadings in Set 4 are zero by definition in the fitted C(*M* − 1) models, due to the first method being selected as reference method in this model.

Figure 5 shows the number of non-significant factor loadings for both trait and method factors. These factor loadings are shown in sets on the *x*-axes, with each set containing three loadings which correspond to the same method. Sets 1–3 show the trait factor loadings (λ), whereas Sets 4–6 show the method factor loadings (γ): Set 1 contains λ_11−31_, Set 2 contains λ_12−32_, Set 3 contains λ_13−33_, Set 4 contains γ_11−31_, Set 5 contains γ_12−32_, and Set 6 contains γ_13−33_. For each set of loadings, the number of loadings (0, 1, 2, or 3) for a specific simulation condition that were not statistically significant at the 0.05 level is reported. Note that for some model conditions, the total number of reported cases is lower than for others. This is due to non-converged models in some of the cells of the simulation design. Also note that the method factor loadings pertaining to Set 4 are always (trivially) non-significant in each fitted C(*M* − 1) model, because these loadings are all set to zero by definition in this model (the first method was selected as reference method in this model; there is no method factor for the reference method). The results shown represent averages across all levels of reliability and sample size for that condition.

Figure 5 shows that for the correctly specified UM_constrained_ and C(*M* − 1) models, all trait and method factor loadings were consistently statistically significant except under the most extreme conditions of either very low or very high consistency in the population model. In these extreme cases, population trait factor loadings (in the case of very low consistency) or population method factor loadings (in the case of very high consistency) were rather small in the population models, lowering the statistical power to detect such loadings as being significantly different from zero in a Monte Carlo sample). In the correctly specified UM_constrained_ case, non-significant loadings occurred only for trait loadings and only in the lowest consistency condition (0.1), in which only 10% of the true score variance were accounted for by the trait and trait factor loadings were accordingly small. All method factor loadings were significant in all conditions in the correctly specified UM_constrained_ case.

In the correctly specified C(*M* − 1) case, the number of non-significant trait loadings was trivially small. As in the correctly specified UM_constrained_ case, non-significant trait loadings occurred only in the lowest consistency conditions. The correctly specified C(*M* − 1) models had more instances of non-significant method factor loadings, but only in the highest consistency condition (0.9), in which 90% of the true score variance was accounted for by the reference trait factor and the population method factor loadings for the non-reference methods were accordingly small. Note that the highest consistency condition was also the only one that produced some convergence problems in the correctly specified C(*M* − 1) cases in the present simulation.

When the C(*M* − 1) model was incorrectly fit to UM_constrained_ population data, all trait and method factor loadings were significant, except in conditions of low consistency; the statistical significance of all method factor loadings was unaffected even in conditions of very high consistency. When the UM_constrained_ model was incorrectly fit to C(*M* − 1) population data, all trait and method factor loadings were significant, except in the highest consistency condition; only in this condition were method factor loadings more frequently found to be non-significant.

The UM_unconstrained_ model fit to UM_constrained_ population data produced mostly significant trait and method factor loadings, except in cases of very low consistency (in which case there was a high rate of non-significant trait factor loadings) and very high consistency (in which case there were several instances of non-significant method factor loadings). When the CM model was fit to UM_constrained_ population data, a high rate of non-significant trait and/or method factor loadings resulted. Non-significant method factor loadings were seen across all levels of consistency, although with a more frequent occurrence for the higher consistency conditions. Non-significant trait factor loadings were mostly observed in conditions of low consistency.

Of particular interest to us were the cases in which either the UM_unconstrained_ or CM models were fit to data generated based on the C(*M* − 1) model. For the UM_unconstrained_ model fit to C(*M* − 1) population data, we found that trait loadings were consistently significant, whereas one or more full sets of method factor loadings were frequently non-significant in the high method factor correlation (0.8) conditions. In the moderate method factor correlation (0.5) condition, this issue occurred only in cases of high consistency. In the low method factor correlation (0.2) condition, the issue was also positively related to trait consistency: the higher the consistency, the more non-significant method factor loadings. At low levels of method factor correlation in the population C(*M* − 1) model, the first method factor set collapsed most often. At medium levels of method factor correlation, the rate of collapse was roughly equal for all method factor sets. At high levels of correlation, the second or third method factor set collapsed most often, whereas the first set of method factor loadings was more stable.

With regard to the CM model, Figure 5 reveals that the CM model's convergence problems were similarly high across all C(*M* − 1) population conditions. Fitting the CM model to C(*M* − 1) data frequently resulted in non-significant method factor loadings. This was particularly the case for C(*M* − 1) population data with a high method factor correlation (0.8). Non-significant trait loadings were less frequently found in the CM model, but did occur when consistency values in the population models were low.

## Discussion

Data structures analyzed by psychologists and other social scientists are often multifaceted. Multifaceted data structures result, for example, when researchers collect multimethod data (a single or multiple traits measured by multiple methods or reporters), longitudinal data (a single or multiple traits measured at multiple time points), or hierarchically structured cross-sectional data (e.g., one general ability factor plus multiple specific factors of intelligence). When analyzing faceted data structures, researchers often assume that some or all observed variables are influenced by at least two latent variables. As a consequence, complex CFA models with general (“trait”) and specific (“method,” “specific,” or “group”) factors are popular and widely used to analyze such data structures. Models in which observed variables load onto multiple latent variables are not without empirical problems, however, as is evidenced by numerous methodological and applied studies.

In the present paper, we used simulations to examine a specific phenomenon that is often observed in empirical applications of MTMM, LST, or bifactor models: A substantial number of applications of models with *M* method or specific factors in addition to a general trait factor have resulted in one or more method (specific) factors that are empirically unstable in terms of a non-significant factor variance or non-significant factor loading parameter estimates. When one of the method (specific) factors collapses empirically, the interpretation of the trait factor as a “general” factor is jeopardized, as the general factor in this case becomes specific to the indicators that have no method factor. This *post-hoc* interpretation is at odds with the *a priori* intended interpretation of this factor as a “general factor” in the UM_constrained_, UM_unconstrained_, and CM models.

One goal of our simulations was to test the hypothesis that a higher rate of unstable method factors may be a result of a mismatch between the measurement design and the fitted model. Specifically, we hypothesized that models with *M* method (or specific) factors may show the above-mentioned problem when they are fit to data generated from a design with structurally different (rather than interchangeable) methods or facets. Results from our simulation provided support for our main hypotheses according to which (1) an incorrect specification of CFA-MTMM models would be difficult to detect based on commonly used model goodness-of-fit criteria and (2) the method factor collapse issue would occur more frequently when the UM_unconstrained_ or CM models are fit to C(*M* − 1) population data. We found that commonly used model fit criteria frequently failed to reveal incorrect model specifications. This was true even for the χ^2^ test at a large sample size of *N* = 1000, at which this test is commonly regarded as a rather sensitive measure of (mis)fit. The only situation in which fit indices were relatively helpful in identifying a model misspecification was when the UM_constrained_ model was incorrectly fit to C(*M* − 1) data. But even in this case, the incorrectly specified UM_constrained_ model was reliably detected by the χ^2^ test only in the condition of a large (0.8) method factor correlation in the data-generating C(*M* − 1) model. In all other cases, commonly used criteria for adequate model fit would not have led to a proper rejection of misspecified models.

One (partial) explanation for the frequent failure of fit criteria to detect misspecified models may be that the UM_unconstrained_ and CM models in the current design with one trait, three methods, and three indicators per TMC use more parameters (i.e., 27 and 30 parameters, respectively) to fit the data than the UM_constrained_ and C(*M* − 1) models, which use only 15 and 25 parameters, respectively. Therefore, the UM_unconstrained_ and CM models overfit data generated by either the UM_constrained_ or C(*M* − 1) models. By using more parameters, these models have more room to fit a range of data structures despite being misspecified. This may explain why fit criteria were rarely able to detect such misspecifications in our simulation. This does not explain, however, why the misspecification of fitting the UM_constrained_ model to C(*M* − 1) data was also difficult to detect in the present simulation, given that the UM_constrained_ model had 10 parameters less than the C(*M* − 1) model in the present study.

Overall, our results show that researchers cannot exclusively rely on fit indices when judging the adequacy of a CFA-MTMM model for a given data set. This is in line with findings of other researchers (e.g., Maydeu-Olivares and Coffman, 2006) who also reported difficulties in distinguishing between different CFA-MTMM models based on fit statistics. This issue may be part of the reason why researchers are often unsure about which CFA-MTMM model to accept and interpret for a given set of data. It is important for researchers to realize that CFA-MTMM models, in particular the UM_unconstrained_ and CM models, are highly parameterized and can therefore fit a wide variety of data well, even data that was generated by a different process. Researchers thus cannot conclude from favorable model fit results alone that the estimated model parameters accurately reflect the MTMM measurement design.

The second important finding was that, as hypothesized, the method factor collapse problem did occur more frequently when data generated from structurally different methods were fit with the UM_unconstrained_ or CM models as compared to correctly specified models. This issue was particularly severe in cases of a high correlation between method factors in the data-generating C(*M* − 1) model. In our experience, high method factor correlations are not uncommon in actual MTMM research, as structurally different methods often share a common perspective that deviates from the reference method perspective (e.g., mother and father reports vs. child self-reports as reference method). These findings thus provide preliminary support for the hypothesis that the commonly seen problem of one method (specific) factor collapsing in models with a general trait and *M* method (specific) factors may occur more frequently when methods (or occasions, facets) are not interchangeable (randomly drawn from a uniform set of methods, occasions, or facets), but rather structurally different.

A likely technical explanation for the method factor collapse problem is that both the UM_unconstrained_ and CM models typically overfit data generated by structurally different methods as explained above [assuming that structurally different methods are most appropriately represented by a C(*M* − 1) data-generating process, which uses fewer parameters in most MTMM designs]. Based on our findings, we recommend that researchers pay careful attention to the measurement design. Were interchangeable (randomly selected) or structurally different (fixed) methods used in the study? Researchers should select models that are appropriate for the measurement design at hand in line with Eid et al. (2008). It is our impression that by using this strategy, researchers can avoid many of the issues related to ambiguous fit results, improper solutions, and the interpretation of potentially spurious method factors.

It appears that structurally different methods are currently much more frequently used in empirical MTMM applications than interchangeable methods. Researchers often apply the UM_unconstrained_ or CM models to structurally different methods. This may explain why collapsing method factors are commonly encountered in such applications. We recommend that researchers working with structurally different methods should consider using the C(*M* − 1) approach rather than the UM_unconstrained_ or CM approaches. In our view, this may be an effective way to avoid the method factor collapse problem and obtain meaningful and easily interpretable results in MTMM studies.

### Implications for longitudinal and bifactor modeling

#### Latent state-trait models

Our findings have implications beyond the MTMM literature, as similarly structured models are widely used in other research contexts as well. In longitudinal studies of state vs. trait components of social science constructs, researchers often apply LST models. Many LST models have a similar structure to the UM-type models, with the uncorrelated method factors replaced by uncorrelated occasion-specific factors. As noted by Geiser et al. (2015) many LST applications use models with unconstrained factor loadings across time. Such unconstrained LST models are thus equivalent in structure to the UM_unconstrained_ model studied in our simulation—a model that fit a wide variety of data well yet showed the factor collapse problem when fit to structurally different methods.

Indeed, the problem of one or more occasion-specific (situation) factors collapsing has been observed in the LST literature as well (Schermelleh-Engel et al., 2004). According to our findings, this problem might be partly explained by the fact that situations may not always be truly selected at random in LST studies. Structural differences between situations can arise, for example, because participants first have to get used to the assessment procedure, often rendering the first measurement occasion “structurally different” from subsequent occasions. For structurally different situations, occasion-specific factors might collapse more frequently. We recommend that researchers routinely specify LST models with time-invariant parameters, leading to a UM_constrained_ structure. This structure is more restrictive than the UM_unconstrained_ structure, and, according to our present simulation, can be more easily falsified by common fit criteria than a UM_unconstrained_ structure. If a UM_constrained_ structure (measurement invariance) cannot be rejected based on the chi-square test, then this provides the researcher with more confidence (albeit not perfect certainty) that the assumption of random situations may be justified. If a UM_constrained_ structure does not hold, then this may be an indication that the chosen situations are not interchangeable and/or that the process is not a simple state variability process, but potentially involves trait changes as well. For cases in which an occasion-specific factor collapses in LST models, Schermelleh-Engel et al. (2004) recommended an approach with *M*−1 occasion-specific factors as a general alternative for LST applications.

#### Bifactor models

Another area of CFA modeling for which our results have implications are bifactor models, which are very popular in research contexts that deal with hierarchically structured or faceted constructs such as intelligence and quality of life. As noted previously, many applications of bifactor models show the issue of one of the *a priori* hypothesized specific factor collapsing. Virtually all applications of bifactor models that we know of leave the factor loadings unconstrained, that is, assume a UM_unconstrained_ structure. This is plausible, because the facets considered in bifactor analyses are often quite diverse. Therefore, researchers do not have a reason to constrain factor loadings in accordance with a UM_constrained_ structure. Hence, researchers often obtain a well-fitting model (because the UM_unconstrained_ model is rather unrestrictive and fits a wide variety of data structures well as demonstrated in the current simulation), yet with partly unstable specific factors. We suspect that the factor collapse issue in applications of bifactor models may be due to the fact that the facets analyzed in bifactor analyses should often be seen as fixed effects rather than as random effects from a measurement theoretical perspective. For example, different facets of quality of life are typically not selected at random from a universe of quality-of-life domains, but are rather fixed. As another example, reasoning, memory, spatial abilities, and mental speed as facets of the general trait “intelligence” are rather fixed as well. The modeling of such “fixed facet” data might therefore also benefit from considering Eid et al.'s (2008) MTMM theory of interchangeable vs. structurally different method designs and corresponding measurement models for structurally different (“fixed”) methods. We suspect that in many cases, the use of an approach with *M*−1 correlated specific factors may lead to more readily interpretable results in applications that to date typically use a bifactor (UM_unconstrained_) approach.

### Limitations and future directions

In the present study, we assumed that we know exactly which type of CFA-MTMM model is implied by measurement designs with interchangeable vs. structurally different methods. In practice, however, we do not know exactly which model is the “true” (data-generating) model for each process. We realize that some MTMM theorists and researchers may question our selection of models chosen to represent interchangeable and/or structurally different data generating processes. For example, Castro-Schilo et al. (2013) argued that the CM model may be most plausible as the data-generating model for MTMM data in general. Nonetheless, we think that our selection of models is at least based on a well-developed measurement theoretical framework of random (interchangeable) vs. fixed (structurally different) methods—whereas other frameworks have not used a measurement theory-driven selection of models at all.

Another limitation of the present study is that we considered only designs with a single trait factor. This was done in order to focus on the specific issue of method factor collapse and to keep the size of the simulation more manageable. In addition, many MTMM applications and most applications of LST and bifactor models use only a single trait, so our findings are informative for these types of applications. Preliminary simulations of our group indicate that for designs with more than one trait factor, goodness-of-fit measures may be somewhat more effective in identifying certain types of misspecified models, although the outcomes were still unsatisfactory. In our simulations, we assumed multivariate normal and complete data. Our findings may not (or only partly) generalize to more realistic conditions of non-normal and/or missing data.

Some researchers might question whether there is a problem at all when a specific factor collapses and argue that this is simply an empirical result (showing that the indicators that were hypothesized to measure this specific factor do not contain specific or method variance, but only “general” trait variance). We argue that the factor collapse issue does pose problems, because it results in *post-hoc* changes in the interpretation of the general factor that are at odds with the *a priori* intended meaning and interpretation of this factor. If one or more specific factors collapse, the general factor becomes specific to the set of indicators for which the specific factor(s) collapsed. The trait can no longer be interpreted as a general factor in the classical sense. Depending on the substantive application, this may or may not pose problems.

In future studies, it would be interesting to determine exactly what mechanisms and conditions result in the method (specific) factor collapse issue in practical applications. Why does this issue occur in some, but not all applications of UM_unconstrained_, LST, and bifactor models? What determines *which* method (specific) factor collapses? (How) should researchers interpret the general trait factor in cases in which one or more specific factors collapse—as a general factor, a specific factor, or not at all? Even though the present simulation provided some evidence that the factor collapse issue may occur more frequently when models with *M* method or specific factors are applied to data that stem from a structurally different method pool, we still do not know what exactly determines when (and which) method or specific factors collapse in MTMM, LST, or bifactor model applications. It will be interesting to study what exactly drives this process and in which way. Future (re)analyses of empirical data, further simulations, or mathematical-theoretical derivations will hopefully shed more light on this issue.

## Funding

This research was funded by a grant from the National Institutes on Drug Abuse (NIH-NIDA), grant #1 R01 DA034770-01 awarded to GL and CG.

### Conflict of interest statement

The authors declare that the research was conducted in the absence of any commercial or financial relationships that could be construed as a potential conflict of interest.

## Footnotes

^1^In the present article, we only consider designs with either interchangeable or structurally different methods, but not the combination of both. Nonetheless, the issues discussed in this paper have direct implications for mixed designs as well.

^2^Note that MI is a necessary but not sufficient condition for the interchangeability of methods. That is, even if MI can be established, methods may still be more adequately classified as structurally different. A sufficient condition for interchangeability is that methods are drawn at random from the same set of methods. In contrast to MI, however, this latter condition is theoretical rather than empirically testable.

^3^To generate some variability in the reliabilities, we used a symmetric unimodal distribution centered at 0 and extending to ±0.025. Specifically, the distribution we used is given by (β_3, 3_ − 0.5)0.05, where β_3, 3_ is the beta distribution of the first kind with both shape parameters equal to 3.
